# The unmet needs of adopting clinical decision support system among physicians in Palestinian hospitals

**DOI:** 10.1371/journal.pone.0310765

**Published:** 2024-12-12

**Authors:** Haya Sultan, Shahenaz Najjar, Faisal Awartani, Hammoda Abu-Odah

**Affiliations:** 1 Department of Pharmacy, Nursing and Health Professions, Birzeit University, Ramallah, Palestine; 2 Faculty of Graduate Studies, Arab American University, Ramallah, Palestine; 3 Department of Public Health and Primary Care, Leuven Institute for Healthcare Policy, KU Leuven, Belgium; 4 UBI Business School, Brussels, Belgium; 5 School of Nursing, The Hong Kong Polytechnic University, Hung Hom, Kowloon, Hong Kong SAR, China; 6 Centre for Advancing Patient Health Outcomes: A JBI Affiliated Group, The Hong Kong Polytechnic University, Hung Hom, Kowloon, Hong Kong SAR, China; 7 WHO Collaborating Centre for Community Health Services, The Hong Kong Polytechnic University, Hung Hom, Kowloon, Hong Kong SAR, China; Tehran University of Medical Sciences, ISLAMIC REPUBLIC OF IRAN

## Abstract

**Introduction:**

Clinical decision support system (CDSS) is an application used to aid decision-making and provide knowledge regarding treatment, diagnosis, and laboratory testing. Despite the associated benefits, the underutilization of CDSS is causing a global challenge. In Palestine, CDSS remains unimplemented, prompting a study on knowledge levels and factors influencing CDSS acceptance among physicians.

**Objective:**

The study aimed to assess physicians’ knowledge of CDSS and understand factors impacting CDSS adoption, including performance expectancy (PE), effort expectancy (EE), computer self-efficacy, involvement in decision-making, perceived threat to autonomy, and social influence.

**Methods:**

A validated questionnaire-based survey consisting of seven constructs was used in this study. The constructs were derived from the unified theory of acceptance and use of technology (UTAUT). The target population was 363 resident physicians and general practitioners (GPs) from three governmental hospitals. In total, 124 participants were enrolled, and data was collected between June 1 and June 30, 2022. Generalized linear regression was used for data analysis.

**Results:**

Most participants were males (75%), with an average of 2.9 years of experience. Despite limited CDSS knowledge, participants strongly intended to adopt it (mean = 3.91, SD = 0.65). Our results showed a significant effect of PE (p = 0.000, r = 0.469), EE (p = 0.001, r = 0.294), computer self-efficacy level (p = 0.015, r = 0.218), perceived threat to professional autonomy (p = 0.001, r = -0.302), and social influence (p = 0.047, r = 0.179) on the intention to adopt CDSS. However, involvement in decision-making about CDSS had no significant impact (p = 0.123, r = 0.140).

**Conclusion:**

This study provides the basis for implementing CDSS in Palestinian settings. Despite the readiness of Palestinian healthcare providers, introducing CDSS as an aiding, effort-free tool is a prerequisite for successful CDSS adoption in Palestine.

## 1. Introduction

One of the most popular emerging technologies in the healthcare sector is the clinical decision support system (CDSS) [1]. CDSS, which is a feature provided by the electronic health record (EHR), is an application that supports clinicians in implementing clinical guidelines at the point of care [2]. It aids decision-making and provides knowledge regarding treatment, diagnosis, and laboratory testing [2]. CDSS plays a significant role in decreasing medical errors and selecting the most appropriate treatment and diagnostic plans for patients, thus reinforcing patients’ safety measures and improving overall quality.

Despite the associated benefits of adopting CDSS, the underutilization of this application poses a global challenge, even for facilities that have already installed CDSS [3–7]. This is likely because successful technology adoption requires users’ acceptance and actual technological use, which surpass the theoretical installation of these systems [8]. Recent research is driven by the need to understand healthcare providers’ perceptions towards CDSS since they play a crucial role in any successful implementation. Physicians, in particular, have negative perceptions of CDSS [9], and the primary barriers to adoption include a loss of professional autonomy, increased medicolegal liability, and a fear of lacking technical capabilities [5, 10].

Even though there hasn’t been much regional research on CDSS, a few studies have looked at this subject from various aspects. Two studies from Saudi Arabia [11, 12] assessed physicians’ perceptions regarding the adoption of CDSS in settings where CDSS has not yet been implemented. According to these investigations, the main obstacles to CDSS adoption were a lack of the necessary computer skills and the fear of losing physicians’ autonomy. The results from these studies supported previous research in which performance expectancy (PE) and effort expectancy (EE) had a significant impact on users’ acceptance. However, social influence did not have an impact on respondents’ intentions to embrace CDSS [11, 12].

Unfortunately, CDSS has not been adopted in Palestinian hospitals yet. Palestinian healthcare providers, particularly those working at governmental hospitals, encounter additional challenges considering their scarcity of resources, fragmented healthcare system, restrictions on movement, and the associated difficulties in recruiting other specialised staff. In the West Bank, there are 2.3 physicians per 1000 population and 1.3 beds per 1000 population [13], while there are 5.32 physicians per 1,000 population in Austria and 3.9 beds per 1,000 population in Australia [9, 12, 14]. According to these figures, the implementation of CDSS can be highly beneficial in Palestinian contexts, as healthcare providers often face numerous cases where any aiding clinical tool can facilitate the daily working clinical environment and subsequently enhance patients’ outcomes.

Applying health information technology (HIT) to enhance healthcare services in Palestine has several hurdles; studying healthcare practitioners’ perceptions will help highlight the reasons why they are hesitant to employ CDSS in healthcare settings. CDSS implementation will not be successful until the obstacles that impede healthcare providers’ readiness are overcome. Due to the occupation, restricted resources, and restrictions on healthcare practitioners’ movement, Palestine has unique obstacles that delay the application of HIT in the healthcare industry. Since Palestinian hospitals joined the World Health Organization (WHO)-led Patient Safety Friendly Hospital Initiative (PSFHI) in 2012 to improve patient safety standards, the need for CDSS has become even more urgent, as the primary goal of CDSS is patients’ safety [15]. All these factors exacerbate the need for CDSS to facilitate decision-making at the point of care. Therefore, this research will be unique and interesting, as limited research is available regarding CDSS in Palestinian hospitals, and it will help identify fears related to adopting CDSS and facilitate overcoming these concerns. Moreover, identifying factors affecting users’ acceptance will help in highlighting strengths and revealing weaknesses that needs to be corrected.

The “unified theory of acceptance and use of technology” (UTAUT), developed in 2003 by Venkatesh and colleagues [16], was used as a base model and modified to achieve the scope of this study. According to the UTAUT model, healthcare providers’ adoption of any technology will be influenced by the following variables: PE, EE, social influence, and facilitating conditions. This model can predict 70% of the intention to use IT, compared to the other previous models that were only able to predict 40% of users’ acceptance, making it the most reliable model to be implicated in the healthcare sector and a strong predictor of users’ IT adoption tendency [3, 5, 7, 8, 16–18]. It is important to note that this is the first study to approach this topic from this angle in Palestine. This study aims to assess the level of knowledge and factors influencing the intention to adopt CDSS in Palestinian settings.

## 2. Methodology

### 2.1 Study participants and sample size

A cross-sectional study design employing a deductive approach was conducted in this research. The study took place at three Palestinian governmental hospitals in the West Bank between June 2022 and August 2022. The researcher obtained approval from the Palestinian Ministry of Health, Arab American University’s research committee (2022/5/10), and the medical directors of the participating facilities. After obtaining ethical approval from the Palestinian Ministry of Health (PMOH), the medical directors of the three hospitals were contacted to determine the size of the target population. All participants were given a written informed consent form along with the questionnaires. This informed consent form explained the purpose of the study, their right to participate, and how their information would be kept confidential. Before answering the questionnaire, all participants gave voluntary consent to participate in this study and abide by the conditions in the consent form. The target group for this study was general practitioners and resident physicians.

The target population was found to be 363 providers. Notably, males accounted for 75% (n = 272) of the target population. The Raosoft online calculator was used to estimate the required sample size using a 95% confidence interval and a 10% margin of error [19]. The calculator suggested a sample size of 77 participants, but a larger sample size of 124 residents or GPs was used in this study. Stratified sampling was utilized to ascertain the male-to-female ratio, ensuring the sample’s representativeness. Consequently, females accounted for 25% of the selected sample size (n = 31). Moreover, any resident doctor working in any department of these governmental hospitals (GP or in a residency program) was eligible to participate, with no eligibility criteria for individual residents’ characteristics.

### 2.2 Conceptual framework and hypothesis

In this study, UTAUT was used as a base model to shape our conceptual framework, which was adjusted to suit the Palestinian context and effectively explain the perception of healthcare providers. Since UTAUT is a general model, we changed it to better address physicians’ individualized characteristics. The original UTAUT model assumes that the intention to adopt any technology is mainly mediated by four parameters: PE, EE, social influence, and facilitating conditions. Current evidence suggests that PE and EE are more strongly correlated to accepting CDSS than the other two parameters (social power and facilitating conditions), which might have a low or insignificant effect. The facilitating conditions construct was specifically found to have the most negligible impact on users’ acceptance [20–24]. Recent research highlighted the importance of considering computer self-efficacy, perceived threat to professional autonomy, and level of involvement in decision-making as potential mediators of users’ acceptance [3, 7, 10, 24]. Therefore, the facilitating conditions construct was replaced by other, more influential variables. Based on the above-suggested parameters, we hypothesize the following:

Hypothesis #1**:** A negative relationship exists between the intention to adopt CDSS and the perceived threat to professional autonomy.
Hypothesis #2: The level of involvement in selecting CDSS is positively linked to the intention to adopt CDSS.
Hypothesis #3: PE has a positive relationship with the intention to adopt CDSS.
Hypothesis #4: EE has a positive relationship with the intention to adopt CDSS.
Hypothesis #5: Social influence positively affects the intention to adopt CDSS.
Hypothesis #6: Computer self-efficacy is linked to a positive intention to adopt CDSS.

### 2.3 Questionnaire instrument

The questionnaire for this study was derived from a survey conducted in Malaysia that looked at a comparable research question [7]. The rationale for choosing this survey stems from the fact that Malaysian doctors and Palestinian doctors have a lot in common. First of all, like Palestine, Malaysia is a CDSS pre-adopter country. Second, statistics indicate that Malaysian doctors face a comparable workload. Malaysia has two physicians per 1,000 population, while Palestine has 2.3. Comparing beds per 1000 population, it can be noted that Palestine has 1.3 beds per 1000 population, whereas Malaysia has 1.98. Therefore, this survey serves as a reasonable tool to benchmark the perceptions of Palestinian physicians regarding CDSS [14, 25–27].

Our survey consists of seven constructs, with a total of 41 items: “intention to adopt CDSS (six items), “PE” (six items), “EE” (six items), perceived threat” (five items), “computer self-efficacy” (eight items), “involvement in decision-making” (four items), and social influence” (six items). A five-point Likert scale was used to answer these measures, where “strongly disagree” = 1 and “strongly Agree” = 5. The questionnaire was translated into Arabic as it is the mother tongue of the participants, and back translation to English was used to ensure maintaining the original meaning.

In order to ensure that the questionnaire content was valid, seven experts were consulted, including two academics, two health informatics researchers, and three physicians. Moreover, Cronbach’s alpha was calculated to check the instrument’s reliability and internal consistency. The alpha value for all constructs exceeded the recommended threshold of 0.7. This indicates that measurement errors were minor [28]. Additionally, this questionnaire was pilot-tested by six resident doctors before it was widely disseminated and was slightly modified accordingly.

### 2.4 Data collection

The first author (HS) personally handed questionnaires to the intended participants and gathered all necessary data. The data was collected between June 1 and June 30, 2022. Most surveys were instantly filled out and immediately returned to the researcher. However, several respondents had long shifts and needed 15 to 60 minutes to complete the questionnaires.

### 2.5 Statistical analysis

The Statistical Package for the Social Sciences (SPSS) version 25 was used for data entry and analysis. Surveys were included in the analysis if at least 75% of the items (at least 30 items) were answered. However, all surveys were deemed usable. The third and fourth items from the intention to adopt construct were reverse coded into the same variables, as they had a negative direction. Furthermore, the mean Likert scale of the following constructs was computed for each questionnaire: a) intention to adopt CDSS b) PE c) EE d) computer self-efficacy e) perceived threat to professional autonomy f) level of involvement in decision-making and g) social influence.

Descriptive statistics were used to describe the respondents’ characteristics and present the mean and standard deviation (SD) of the study’s seven constructs. Additionally, independent sample t-test and ANOVA were used as appropriate to explore the association between the intention to adopt CDSS and demographic factors. Pearson correlation was also used to identify the direction and relation between continuous factors. Factors with a significant level p < 0.05 in the bivariate analysis and Pearson correlation were included in the generalized linear regression model (GLM) for the purpose of identifying the factors associated with the intention to adopt CDSS. All statistical tests were two-tailed, and p values of less than 0.05 were considered significant.

## 3. Results

### 3.1 Participants characteristics

A total of 124 resident doctors and GPs working at three different governmental hospitals participated in this study. The participants’ sociodemographic characteristics, as displayed in Table 1, indicate that the majority of participants (n = 93, 75%) were males. The mean working experience was 2.9 years, with one year being the minimum experience and 15 years being the highest. Regarding CDSS usage and familiarity, most participants reported low to moderate previous knowledge of CDSS, while 89.5% had not used a CDSS before.

**Table 1:** Characteristics of the participants.

Characteristic	Categories	Number (n)	Percent (%)
Gender	Female	31	75%
	Male	93	25%
Age	20–29	98	79%
	30–39	21	16.9%
	40–49	5	4%
Years of experience**	1–4	87	70.2%
	5–10	15	12%
	11–15	6	4.8%
Specialty*	Internal	18	14.5%
	Pediatrics	23	18.5%
	Radiology	1	0.80%
	Gynecology	14	11.3%
	Surgery	12	9.7%
	Oncology	3	2.4%
	Other	51	41.1%
Previous CDSS	Very high	5	4%
knowledge	High	15	12.1%
	Moderate	47	37.9%
	Low	33	26.6%
	Very low	24	19.4%
Previous CDSS use	Yes	11	8.9%
	No	111	89.5%

* Missing less than 5%;

** Missing less than 15%.

### 3.2 Descriptive statistics

The mean scores, SD, and correlation coefficients among the study constructs are presented in Table 2. Interestingly, our results showed a high intention of Palestinian healthcare providers to adopt CDSS, with a mean of 3.91±0.65. Providers also confirmed having a good influence on their colleagues, with a mean of 3.96±0.57. Also, they believe having a CDSS will enhance their performance and will be easy to use (3.82±0.67 and 3.86±0.66, respectively). Additionally, respondents believe that they have good computer skills and regard the CDSS as posing a modest threat to them.

**Table 2:** Descriptive statistics (mean and SD) and correlations across constructs.

Construct	Mean (SD)	1	2	3	4	5	6	7
		Correlation						
1. Intention to adopt CDSS	3.91±0.65	1	0.467**	0.294**	-0.302**	0.218*	0.140	0.179*
2. PE	3.82±0.67		1	0.620**	-0.195*	0.414**	0.442**	0.469**
3. EE	3.86±0.66			1	-0.172	0.454**	0.355**	0.515**
4. Perceived threat	3.19±0.67				1	-0.129	-0.107	-0.129
5. Computer self-efficacy	3.88±0.61					1	0.306**	0.468**
6. Involvement in decision-making	3.22±0.90						1	0.388**
7. Social influence	3.96±0.57							1

PE-performance expectancy, EE-effort expectancy, SD- standard deviation, CI- confidence interval

** Correlation is significant at the 0.01 level

* Correlation is significant at the 0.05 level

### 3.3 Factors affecting the intention to adopt CDSS

Our findings showed that the intention to adopt CDSS was significantly and positively associated with the following variables: PE (r = 0.469, p = 0.000), EE (r = 0.294, p = 0.001), healthcare professionals’ computer self-efficacy (r = 0.218, p = 0.015), social influence (r = 0.179, p = 0.047). While there is a statistically significant negative relationship with perceived threat to professional autonomy (r = -0.302, p = 0.001), no association was found with the level of involvement in decision-making (r = 0.140, p = 0.123) (Table 2). Thereby, five of our suggested hypotheses were confirmed (hypothesis # 1, 3, 4, 5, 6) as the corresponding p-values were < 0.05, while only one hypothesis regarding involvement in decision-making was rejected (hypothesis #2), as the p-value was > 0.05.

The factors with a significant level p <0.05 in the bivariate analysis (Table 2 and S1 Table) were included in the GLM, which was utilized to predict factors influencing the intention to adopt CDSS. The findings showed that PE (β = 0.434; *p* value = 0.000) and perceived threat (β = -0.216; *p* value = 0.005) were independently associated with the intention of healthcare providers to adopt CDSS (Table 3).

**Table 3:** Generalized linear regression model for factors influencing providers’ intention to adopt CDSS.

Variable	β	SE	95% CI	Wald	P
PE	0.434	0.99	0.24–0.62	19.14	***0*.*000***
EE	0.002	0.104	-0.20–0.20	0.001	*0*.*981*
Perceived threat	-0.216	0.76	-0.36- -0.06	7.88	***0*.*005***
Computer self-efficacy	0.045	0.09	-0.14–0.23	0.213	*0*.*644*
Social influence	-0.089	0.108	-0.32–0.12	0.663	*0*.*409*

PE-performance expectancy, EE-effort expectancy,

Bold indicates significant level

Moreover, our results didn’t reveal any association between sociodemographic characteristics and the intention to adopt CDSS. As shown in S1 Table, for all the studied variables, p-values were > 0.05, indicating that CDSS adoption is not influenced by demography.

## 4. Discussion

This study revealed a very low to moderate level of general understanding of CDSS among the study participants, similar to other developing countries considered pre-adopters like Palestine [7, 29].

There was no evidence that sociodemographic characteristics directly correlated with the intention to adopt CDSS. Our results are in line with other studies, which revealed that professional traits such as decision-making authority, technological proficiency, and confidence in one’s clinical judgment had a more substantial influence on the intention to adopt CDSS than demographic factors like age, gender, and specialization [7, 19, 28–30].

Additionally, our study identified important factors significantly related to the intention to adopt CDSS. Similar to our results, PE and EE were identified by other studies as essential factors influencing the readiness to use CDSS. It is reasonable to think that when somebody perceives that a system will be helpful in their daily work and ensure ease of use, they will be more open to using it [3, 5, 20, 22, 23, 30–35].

Since PE and EE play a vital role in successfully using CDSS, policymakers are encouraged to highlight the added value of incorporating CDSS in Palestinian settings and its positive contribution to patient outcomes. Ensuring users that the system will be effort-free is another vital variable to consider. Stakeholders should implement CDSS only after a proper and careful selection of its features. According to the available literature, alert fatigue and the associated troublesome use of the system have been identified as the primary concerns of physicians in some settings [11, 35]. Additionally, a recent study highlighted that implementing CDSS in developing countries might be even more challenging due to the limited IT solutions and decreased computer literacy among physicians [36]. Therefore, careful CDSS selection in collaboration with expert IT companies and physician involvement is essential for successful implementation in Palestinian contexts.

Regarding computer self-efficacy, other studies demonstrated that computer self-efficacy significantly influenced the intention to use CDSS, which is consistent with our findings [3, 36] As noted earlier, we updated our model to include this variable; as a result, future studies are encouraged to address it as well, given the noteworthy impact we discovered on the intention to adopt CDSS.

Furthermore, our findings highlighted a crucial problem: the need for more Palestinian doctors’ participation in decision-making. Other developing nations, besides Palestine, appear to be facing the same problem [3]. Additional attention should be attributed to hospital physicians, as they are the pillars of these organizations. Similarly, recent studies have also suggested and urged the involvement of physicians in the decision-making process, due to the advantages it offers for a smoother implementation of CDSS [32].

As observed in the results section, our hypothesis regarding perceived threat to professional autonomy was verified, revealing a robust inverse association. Even though it wasn’t part of the original UTAUT model and was added to our conceptual framework, this variable and PE impacted users’ acceptance the most. As the fear of losing control over the profession increases, the intention to use the system decreases. This relationship was highly studied in the context of CDSS adoption. The same negative relationship was identified by several studies [3, 37–39]. New technology may cause doctors to feel threatened for several reasons. First, some people think that CDSS may eventually fill their positions and replace them. Second, some perceive it could disturb the workflow and decrease clinicians’ time with their patients. Third, some physicians think their knowledge loses its flexibility, and the interventions restrict their clinical judgments [7]. This finding necessitates careful planning and involving healthcare providers in the selection process. To ensure that CDSS is successfully adopted, stakeholders must ensure clinicians know how their professional roles are acknowledged and reinforced by utilizing CDSS, rather than the contrary.

Social influence was the final factor influencing Palestinian doctors’ intentions to adopt CDSS. As healthcare professionals urge one another to take the same actions, our theory about how well social interactions boost the intention for adoption is proven to be accurate, confirmed by previous studies [18]. However, another two studies found contradictory results regarding the social influence variable [3, 11]. The social influence could be related to cultural differences, which justifies the conflicting results. However, since we identified this variable, it is worth considering when applying CDSS in Palestinian contexts.

Notably, this study is the first to tackle this issue in Palestine. Hopefully, this paper will be able to fill the existing gap and contribute to a better comprehensive understanding of the current perceptions regarding CDSS adoption by Palestinian healthcare providers, which can be used later as a framework for proposing a suitable CDSS and paving the way for the proper and practical implementation of CDSS across Palestinian hospitals.

## 5. Strengths

Our study is the first to address CDSS in Palestine from this perspective. Also, no earlier information was available about any potential factors that influence the desire to adopt CDSS in Palestinian contexts. Last but not least, the Palestinian resident/GP was the core of the study, which enhances the significance of this research given that they are the key players in successful implementation.

## 6. Limitations

Only three governmental hospitals were included due to the study’s limited resources. As a result, the findings of this study cannot be extrapolated to the private sector. Second, the study’s cross-sectional design seeks out prevalence and associations rather than demonstrating causal relationships.

## 7. Practical implications for the Palestinian healthcare sector

Our study has several implications for the Palestinian healthcare sector, including but not limited to the following: this study successfully identified potential determinants for CDSS adoption in Palestine, including the importance of PE, EE, computer self-efficacy, social influence, and perceived threat to professional autonomy on users’ acceptance of CDSS. Therefore, managers and stakeholders are encouraged to inform medical staff about the potential benefits of CDSS and ensure that a CDSS is an aiding tool rather than a threat to their autonomy, as PE and perceived threat showed the highest effect on users’ acceptance. The PMOH may conduct a number of training sessions to provide doctors with the abilities they need to get past their prejudices about technology. The PMOH is also encouraged to send a number of physicians abroad to settings where CDSS was successfully implemented to reflect the positive impact of this tool. Additionally, focusing on the social environment is a key step in the planning phase as well.

## 8. Conclusion

Despite the unfamiliarity with CDSS, a high intention to adopt CDSS was present among Palestinian physicians. Implementing CDSS in Palestine can be a challenging task. However, this study identified important factors that affect users’ acceptance that need to be considered in the planning phase of CDSS.

## Supporting information

S1 Table Bivariate analysis between intention to adopt CDSS and participants’ characteristics. (DOCX)
