# Supplementary material for: The unmet needs of adopting clinical decision support system among physicians in Palestinian hospitals
Source: PLoS One. 2024 Dec 12;19(12):e0310765. doi: 10.1371/journal.pone.0310765 (PMC11637302; doi:10.1371/journal.pone.0310765)
Supplement: S1 Table — (DOCX) [file pone.0310765.s001.docx]

**Supporting Information**

**S1 Table. Bivariate analysis between intention to adopt CDSS and participants’ characteristics**

| Variable |  | Mean | SD | 95% CI | P value |
| --- | --- | --- | --- | --- | --- |
| Age | 20-29 | 3.91 | 0.68 | 3.77-4.05 | 0.998 |
|  | 30-39 | 3.92 | 0.58 | 3.65-4.18 |  |
|  | 40-49 | 3.90 | 0.49 | 3.28-4.51 |  |
| Gender | Female | 4.03 | 0.57 | -0.11-0.42 | 0.255 |
|  | Male | 3.87 | 0.68 |  |  |
| Specialty | Internal | 3.75 | 0.55 | 4.02-2.33 | 0.601 |
|  | Pediatrics | 4.04 | 0.52 | 4.27-3.33 |  |
|  | Gynecology | 3.98 | 0.65 | 4.36-3.00 |  |
|  | Surgery | 4.05 | 0.53 | 4.39-3.00 |  |
|  | Other | 3.89 | 0.74 | 4.09-2.33 |  |
| Experience years | 1-4 | 3.98 | 0.68 | 3.84-4.13 | 0.765 |
|  | 5-10 | 3.91 | 0.58 | 3.58-4.23 |  |
|  | 11-15 | 3.80 | 0.49 | 3.28-4.32 |  |
| Previous CDSS knowledge | High | 4.18 | 0.67 | 3.86-4.50 | 0.136 |
|  | Moderate | 3.87 | 0.58 | 3.70-4.04 |  |
|  | Low | 3.85 | 0.69 | 3.66-4.03 |  |
| Previous CDSS use | Yes | 4.00 | 0.56 | -0.33-0.47 | 0.725 |
|  | No | 3.92 | 0.65 |  |  |
